# Investigation of a MMP-2 Activity-Dependent Anchoring Probe for Nuclear Imaging of Cancer

**DOI:** 10.1371/journal.pone.0102180

**Published:** 2014-07-10

**Authors:** Takashi Temma, Hirofumi Hanaoka, Aki Yonezawa, Naoya Kondo, Kohei Sano, Takeharu Sakamoto, Motoharu Seiki, Masahiro Ono, Hideo Saji

**Affiliations:** 1 Department of Patho-Functional Bioanalysis, Graduate School of Pharmaceutical Sciences, Kyoto University, Sakyo-ku, Kyoto, Japan; 2 Department of Molecular Imaging and Radiotherapy, Graduate School of Pharmaceutical Sciences, Chiba University, Chuo-ku, Chiba, Japan; 3 Radioisotopes Research Laboratory, Kyoto University Hospital, Faculty of Medicine, Kyoto University, Sakyo-ku, Kyoto, Japan; 4 Division of Cancer Cell Research, Institute of Medical Science, The University of Tokyo, Minato-ku, Tokyo, Japan; Faculté de médecine de Nantes, France

## Abstract

**Purpose:**

Since matrix metalloproteinase-2 (MMP-2) is an important marker of tumor malignancy, we developed an original drug design strategy, MMP-2 activity dependent anchoring probes (MDAP), for use in MMP-2 activity imaging, and evaluated the usefulness of this probe in *in vitro* and *in vivo* experiments.

**Methods:**

We designed and synthesized MDAP_1000_, MDAP_3000_, and MDAP_5000_, which consist of 4 independent moieties: RI unit (^111^In hydrophilic chelate), MMP-2 substrate unit (short peptide), anchoring unit (alkyl chain), and anchoring inhibition unit (polyethylene glycol (PEGn; where n represents the approximate molecular weight, n = 1000, 3000, and 5000). Probe cleavage was evaluated by chromatography after MMP-2 treatment. Cellular uptake of the probes was then measured. Radioactivity accumulation in tumor xenografts was evaluated after intravenous injection of the probes, and probe cleavage was evaluated in tumor homogenates.

**Results:**

MDAP_1000_, MDAP_3000_, and MDAP_5000_ were cleaved by MMP-2 in a concentration-dependent manner. MDAP_3000_ pretreated with MMP-2 showed higher accumulation in tumor cells, and was completely blocked by additional treatment with an MMP inhibitor. MDAP_3000_ exhibited rapid blood clearance and a high tumor accumulation after intravenous injection in a rodent model. Furthermore, pharmacokinetic analysis revealed that MDAP_3000_ exhibited a considerably slow washout rate from tumors to blood. A certain fraction of cleaved MDAP_3000_ existed in tumor xenografts *in vivo*.

**Conclusions:**

The results indicate the possible usefulness of our MDAP strategy for tumor imaging.

## Introduction

Tumor metastasis occurs when a subset of tumor cells acquires the ability to break through the basement membrane and invade through dense networks of interstitial extracellular matrix (ECM) proteins [1]. Matrix metalloproteinases (MMPs) constitute the largest family of enzymes responsible for degrading these various ECM components. Since MMP-2 is currently recognized as the subtype that has the best-established association with tumor malignancy [2], *in vivo* imaging of its activity should be useful for tumor diagnosis. Thus, we aimed to develop a novel nuclear imaging probe capable of estimating *in vivo* MMP-2 activity with Single Photon Emission Computed Tomography (SPECT).

**Figure 1:**
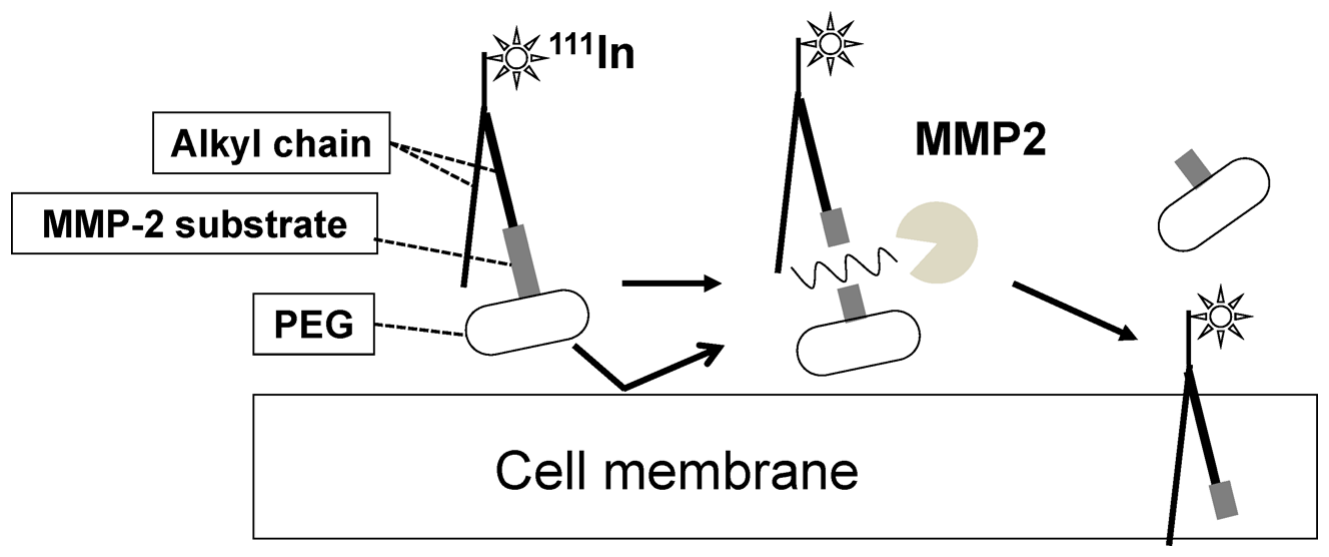
Concept of a novel drug design strategy for a MMP-2 activity-dependent anchoring probe (MDAP).

We originally developed a novel probe design strategy that uses a MMP-2 activity dependent anchoring probe (MDAP) (Fig. 1) to detect MMP-2 activity effectively. Following this MDAP strategy, the probe was expected to be cleaved by MMP-2 enzymatic activity in the vicinity of the tumor, and efficiently trapped in proximal tumor cells. Thus, the radioactivity level detected by SPECT could be correlated with MMP-2 activity in tumors. In this study, we specifically designed and synthesized MDAP_1000_, MDAP_3000_, and MDAP_5000_, consisting of a RI unit (^111^In DTPA), a MMP-2 substrate unit (short peptide) [3], an anchoring unit (alkyl chains) [4], and an anchoring inhibition unit (polyethylene glycol (PEGn; where n indicates the approximate molecular weight, n = 1000, 3000, and 5000) (Table 1). MDAP_CV_, which lacks the PEG moiety, served as a control. We evaluated the feasibility of this drug design strategy and the usefulness of the probes *in vitro* and *in vivo*.

**Table 1:** Probes evaluated in this study.

MDAP_1000_	Palmitoyl-Dap(*p*-SCN-Bn-DTPA-^111^In)-10-Adc-Gly-Pro-Leu-Gly-Val-Arg-Gly-Lys(ivDde)-PEG_1000_
MDAP_3000_	Palmitoyl-Dap(*p*-SCN-Bn-DTPA-^111^In)-10-Adc-Gly-Pro-Leu-Gly-Val-Arg-Gly-Lys(ivDde)-PEG_3000_
MDAP_5000_	Palmitoyl-Dap(*p*-SCN-Bn-DTPA-^111^In)-10-Adc-Gly-Pro-Leu-Gly-Val-Arg-Gly-Lys(ivDde)-PEG_5000_
MDAP_CV_	Palmitoyl-Dap(*p*-SCN-Bn-DTPA-^111^In)-10-Adc-Gly-Pro-Leu-Gly-OH

Dap: 2,3-diamino propionic acid, 10-Adc: 10-amino-decanoic acid, PEG: polyethylene glycol.

## Materials and Methods

### Ethics statement

The animal experiments were conducted in accordance with institutional guidelines and approved by the Kyoto University Animal Care Committee (Permit Number: 2012-49, 2013–33). All surgery was performed under isoflurane anesthesia, and all efforts were made to minimize suffering.

### General

Amino acid derivatives were purchased from Watanabe Chemical Industries (Hiroshima, Japan) and Iris Biotech GmbH (Marktredwitz, Germany). Matrix Assisted Laser Desorption/Ionization-Mass Spectrometry (MALDI-MS) was performed with an AXIMA-CFR Plus apparatus (Shimadzu Corporation, Kyoto, Japan). Reverse Phase High Performance Liquid Chromatography (RP-HPLC) was performed using a Shimadzu-HPLC-gradient system (LC-20AD; Shimadzu Corporation) equipped with a COSMOSIL 5C18-AR-II column (10×250 mm, Nacalai Tesque, Inc., Kyoto, Japan). Products were eluted with a linear gradient starting at 50% solvent B that increased to 80% over 15 min (solvent A: 0.1% trifluoroacetic acid in water [v/v]; solvent B: 0.1% trifluoroacetic acid in acetonitrile [v/v]) at a flow rate of 4.0 ml/min. For tumor metabolite analysis, products were eluted with a linear gradient starting at 50% solvent B that increased to 80% over 30 min at a flow rate of 2.0 ml/min.

### Preparation of probes (Table 1)

Fmoc-Dap(Boc)-10-Adc-Gly-Pro-Leu-Gly-wang-resin (Dap: 2,3-diamino propionic acid, 10-Adc: 10-amino-decanoic acid) was synthesized by a Fmoc-solid-phase peptide synthesis procedure using a peptide synthesizer (PSSM-8; Shimadzu Corporation) with *N*,*N′*-diisopropylcarbodiimide (1.2 eq), *N*,*N*-diisopropylethylamine (1.0 eq), and 1-hydroxylbenzotriazole (1.0 eq) as reagents [5]. After Fmoc group removal with 20% piperidine in *N*,*N*-dimethylformamide, palmitic acid was reacted in a similar way above to yield palmitoyl-Dap(Boc)-10-Adc-Gly-Pro-Leu-Gly-wang-resin. Palmitoyl-Dap(Boc)-10-Adc-Gly-Pro-Leu-Gly-Val-Arg(pbf)-Gly-Lys(ivDde)-PEG_n_-amide resin (n = 1000, 3000, or 5000) was supplied by Scrum Inc. (Tokyo, Japan). Peptide deprotection and cleavage from the resin were simultaneously performed using trifluoroacetic acid/ethanedithiol/water/triisopropylsilane (95/2.5/2.5/1, v/v). The crude peptides were purified by RP-HPLC, followed by lyophilization. The purified compound was reacted with *p*-SCN-Bn-DTPA (2 eq) in *N*,*N*-dimethylformamide (1 ml) and *N*,*N*-diisopropylethylamine (20–100 µl) to adjust the pH of a solution (pH>6), and incubated at room temperature overnight. The radiolabeling precursors were purified by RP-HPLC and characterized by mass spectrometry. [^111^In]InCl_3_ (5.55 MBq in 100 µl of HCl solution, Nihon Medi-Physics Co., Ltd., Japan) was added to each purified precursor (2 nmol) in 0.1 M acetate buffer (pH 6.0, 50 µl) and the mixture was incubated at room temperature for 30 min. Radiochemical purity was estimated by RP-HPLC equipped with a radioactivity detector.

### Measurement of partition coefficients

The experimental determination of probe partition coefficients (log P values) was performed in 1-octanol and 0.02 M phosphate buffer pH 7.4 where the two phases were pre-saturated with each other. 1-Octanol (200 µl) and phosphate buffer (200 µl) were pipetted into LoBind Eppendorf tubes containing 0.37 MBq of probes. The tube was vortexed for 10 seconds, and centrifuged (5 min, 4000×*g*). Aliquots (50 µl) from the 1-octanol and buffer phases were transferred into two test tubes for radioactivity counting with a NaI well-type scintillation counter (1470 WIZARD, Perkin Elmer, Kanagawa, Japan). The partition coefficient was calculated using the equation P =  (counts/µl in 1-octanol)/(counts/µl in buffer).

### Cleavage assay

Human-recombinant MMP-2 protein (902-MP-010, R & D Systems, Inc., Minneapolis, MN USA) was activated with *p*-aminophenylmercuric acetate (1 mM) in assay buffer (50 mM Tris-HCl, 150 mM NaCl, 10 mM CaCl_2_, 5 µM ZnCl_2_, 0.02% Brij 35, pH 7.5). MDAP_1000_, MDAP_3000_, and MDAP_5000_ (370 kBq) were incubated at 37°C for 2 hr with activated MMP-2 (1–5 nM) and a MMP inhibitor (0 or 100 µM, GM6001, Merck KGaA, Darmstadt, Germany). The reaction was quenched by addition of methanol, and the percentage of cleaved peptide was estimated by RP-HPLC.

### Cellular uptake study

HT1080 human fibrosarcoma cells were purchased from American Type Culture Collection (ATCC, Manassas, VA USA) and cultured in DMEM with low glucose (Invitrogen), 10% fetal bovine serum (FBS), and penicillin/streptomycin. MMP-2 cDNA was cloned into the pENTR vector (Invitrogen) and inserted into the pLenti6 vector (Invitrogen) as previously described [6], [7]. For the autoactivated MMP-2 mutant, the sequence that encodes the furin cleavage site of MT1-MMP (Gly-Ala-Glu-Ile-Lys-Ala-Asn-Val-Arg-Arg-Lys-Arg) was inserted between Asn109 and Tyr110 in wild-type MMP-2 by PCR. Lentiviral vectors were prepared and transduced into HT1080 cells as previously described [6], [7]. After overnight preincubation of 5×10^5^ HT1080 cells in FBS-free-DMEM in clean LoBind Eppendorf tubes, cells were incubated with MDAP_CV_, MDAP_1000_, MDAP_3000_ or MDAP_5000_ (37 kBq). Thirty min after the initiation, the cells were washed twice with phosphate buffered saline (PBS) (−) (Seikagaku Biobusiness Co., Japan), transferred to another tube, lysed with 0.2 M NaOH, and the radioactivity counted with a NaI well-type scintillation counter. Protein quantitation was performed by BCA protein assay (Thermo Fisher Scientific, Inc. Rockford, IL USA). The cellular uptake study of MDAP_3000_ pretreated with activated MMP-2 (63 nM) in the presence or absence of GM6001 (100 µM) was performed as described above.

### Preparation of tumor-bearing mice

Female Balb/c *nu-nu* mice (5 weeks old, Japan SLC, Inc., Shizuoka, Japan) were housed under a 12-h light/12-h dark cycle and given free access to food and water. HT1080 cells (5×10^6^ cells/100 µl PBS (−)/mouse) were subcutaneously inoculated into the right hind leg of Balb/c *nu-nu* mice. Animals were used for experiments two weeks after inoculation when the mean tumor size was 5.7±2.2 mm along the major axis.

### In vivo study

MDAP_1000_, MDAP_3000_ and MDAP_CV_ (37 kBq, 100 µl in PBS including 3% bovine serum albumin and 0.1% Tween 80) were injected intravenously into the tail vein of tumor bearing mice. The mice were sacrificed at various post-injection time points (n = 3 for each time point), and the organs of interest including the tumor tissues were collected for determination of the weights. The radioactivity of each sample was measured with a NaI well-type scintillation counter. From fitting to the two phase decay curves for blood radioactivity data analyzed by GraphPad Prism 6 (GraphPad Software, San Diego, CA), whole body pharmacokinetic parameters such as blood half-lives, distribution volume, mean residence time and total clearance were calculated for each of the probes and the values compared. Simple pharmacokinetic analysis using a single tissue compartment model was applied to the biodistribution data to calculate rate constants (K1 and k2) for radioactivity transfer from blood to tumor and clearance from tumor to blood by PMOD version 3.2. In addition, MDAP_3000_ (7.4 MBq in 200 µl) was injected intravenously into the tail vein of tumor bearing mice for metabolite analysis in tumors excised 3, 6, and 24 hr post injection (n = 2 each). Tumor homogenates were prepared on ice and insoluble material was removed by centrifugation after methanol treatment. The resulting supernatant was analyzed by RP-HPLC.

The above data suggested that MDAP_3000_ underwent intratumoral cleavage to some extent after intravenous injection. Thus, for precise analysis of the MDAP_3000_ metabolite generated by MMP activity in tumors, an intratumoral probe administration method was adopted that avoids the possibility that any metabolite made in other tissues would re-distribute to tumors, which is an inevitable issue in intravenous administration methods. MDAP_3000_ (37 kBq, 10 µl in saline) was intratumorally administered to tumor-bearing mice 30 min after intratumoral injection of GM6001 (100 µM, 20 µl in 1% DMSO saline) or 1% DMSO saline (20 µl). Thirty min later, the mice were sacrificed (n = 3 each), the tumors immediately removed and tumor homogenates were prepared on ice. Insoluble material was removed by centrifugation and the resulting supernatant was analyzed by RP-HPLC for cleavage estimation.

Zymography was performed with excised HT1080 tumor homogenates using a Novex Zymogram Gel (Life Technologies Corporation, Carlsbad, CA USA) following the manufacturer' protocol.

The effective dose of MDAP_3000_ was estimated using the area under the non-decay-corrected time radioactivity curves generated from the biodistribution data for each organ with the standard quantitation platform included in Organ Level Internal Dose Assessment software (OLINDA, Vanderbilt University) [8].

### Statistical analysis

Data are presented as mean ± SD. Statistical analysis was performed with the Bonferroni's Multiple Comparison test or unpaired *t* test. A two-tailed value of *P*<0.05 was considered statistically significant.

## Results

### Preparation of probes

**Figure 2:**
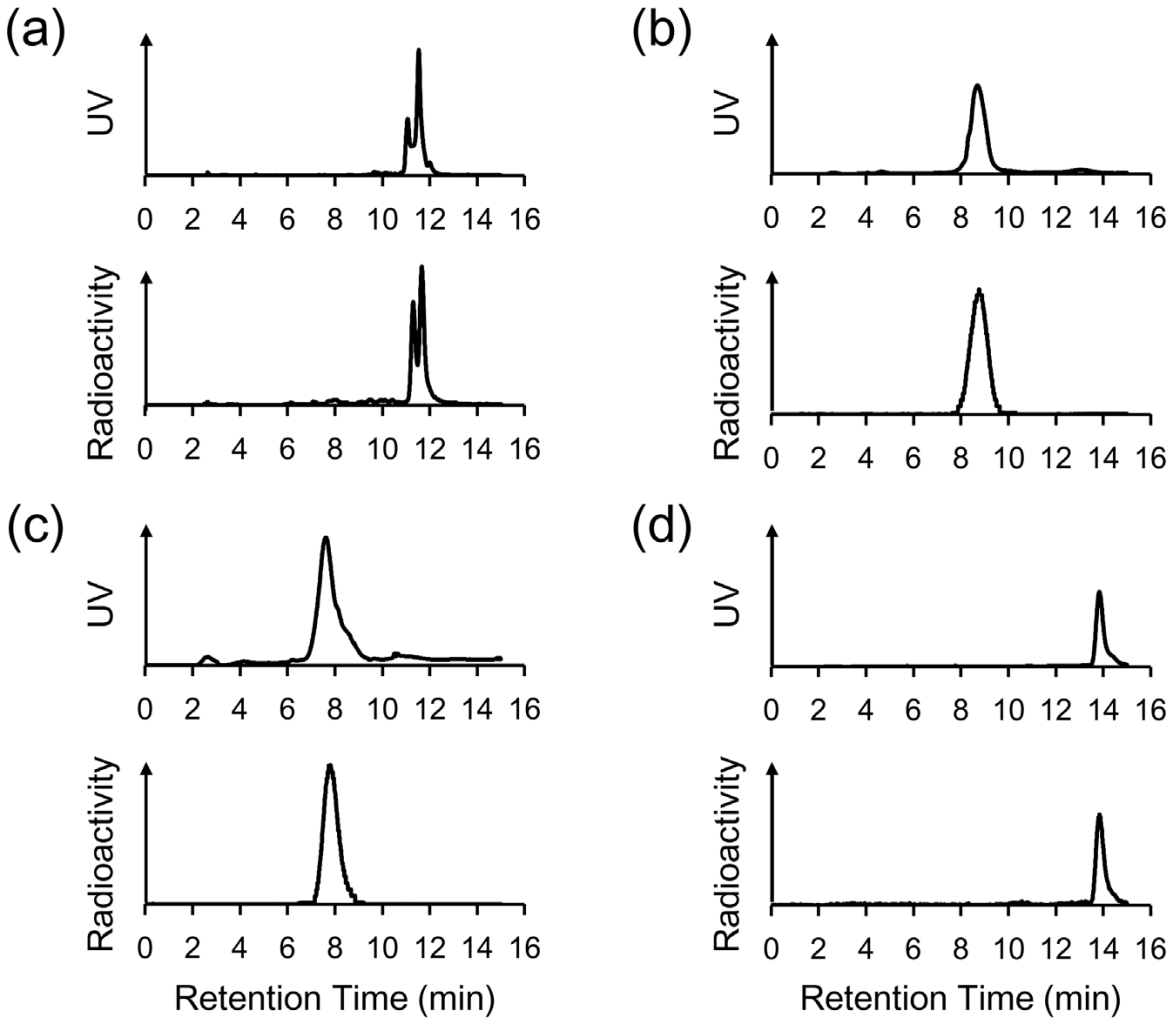
Reverse phase HPLC analysis after probe radiosynthesis. RI charts of (a) MDAP_1000_, (b) MDAP_3000_, (c) MDAP_5000_, and (d) MDAP_CV_ are shown with UV charts of corresponding nonradioactive compounds.

MDAP_1000_, MDAP_3000_, MDAP_5000_ and MDAP_CV_ were obtained with greater than 95%, 97%, 95%, and 98% radiochemical purity, respectively (Fig. 2). The specific radioactivity of the probes was estimated to be 2.8 MBq/nmol, which was dependent on the radioactivity of [^111^In]InCl_3_ used for the radiolabeling procedure. The log P values of MDAP_1000_, MDAP_3000_, MDAP_5000_ and MDAP_CV_ were −0.56±0.13, −0.95±0.12, −1.04±0.16 and 0.29±0.07, respectively.

### In vitro study

**Figure 3:**
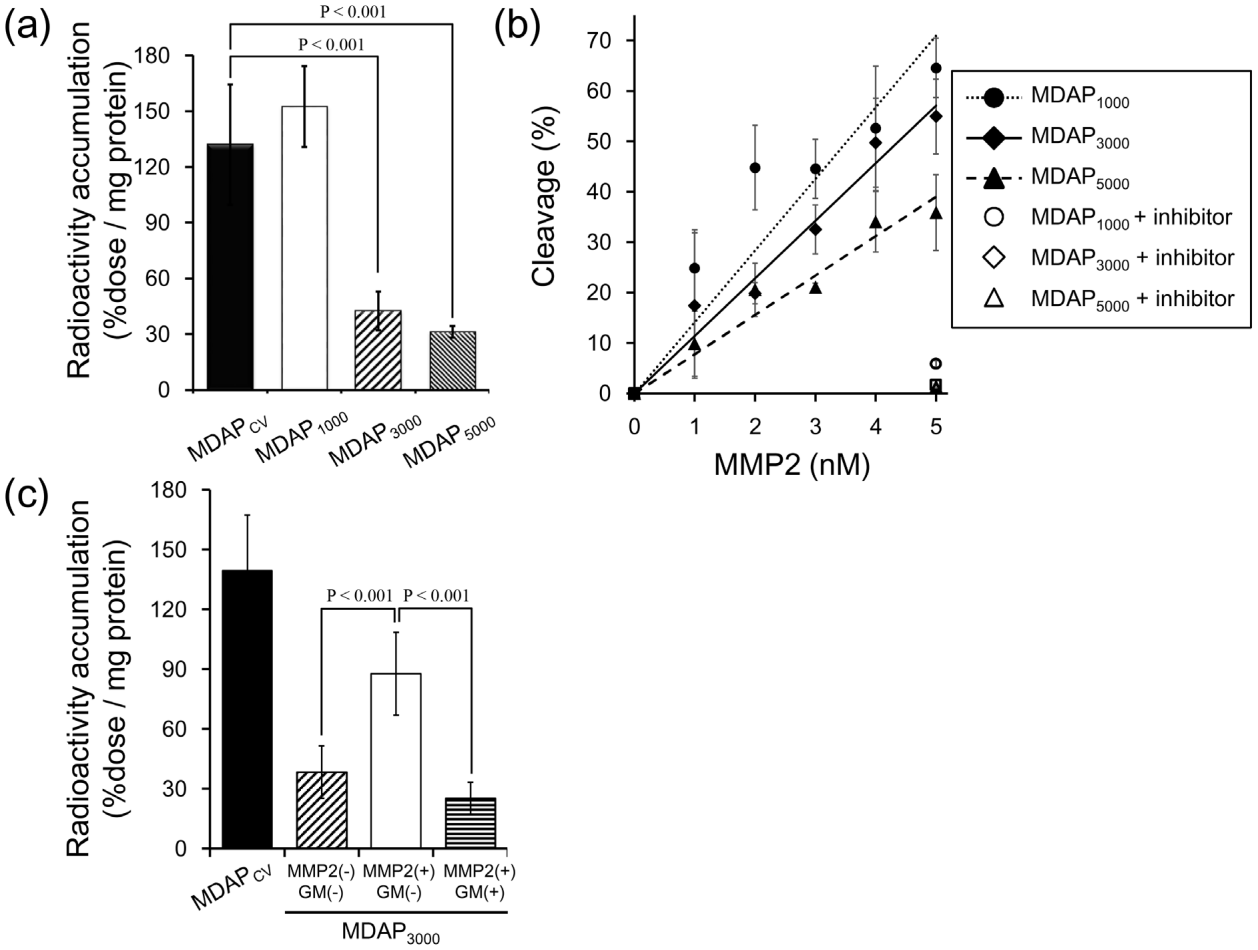
*In vitro* experiment. (a) Cellular accumulation of MDAP_1000_, MDAP_3000_, MDAP_5000_ and MDAP_CV_ estimated by radioactivity counting. (b) Cleavage (%) of MDAP_1000_, MDAP_3000_, and MDAP_5000_ arising from MMP-2 treatment. Values were estimated by reverse phase HPLC. (c) Cellular accumulation of MDAP_3000_ with or without MMP-2 protein and MMP inhibitor (GM6001).

As shown in Fig. 3a, MDAP_1000_ showed high radioactivity accumulation in tumor cells and levels that were similar to MDAP_CV_, while MDAP_3000_ and MDAP_5000_ showed significantly lower radioactivity. MMP-2 cleaved MDAP_1000_, MDAP_3000_ and MDAP_5000_ in a concentration-dependent manner (Fig. 3b). MDAP_3000_ pretreated with MMP-2 accumulated in cells to higher levels, while additional treatment with a MMP inhibitor completely blocked this increased uptake (Fig. 3c).

### In vivo study

**Figure 4:**
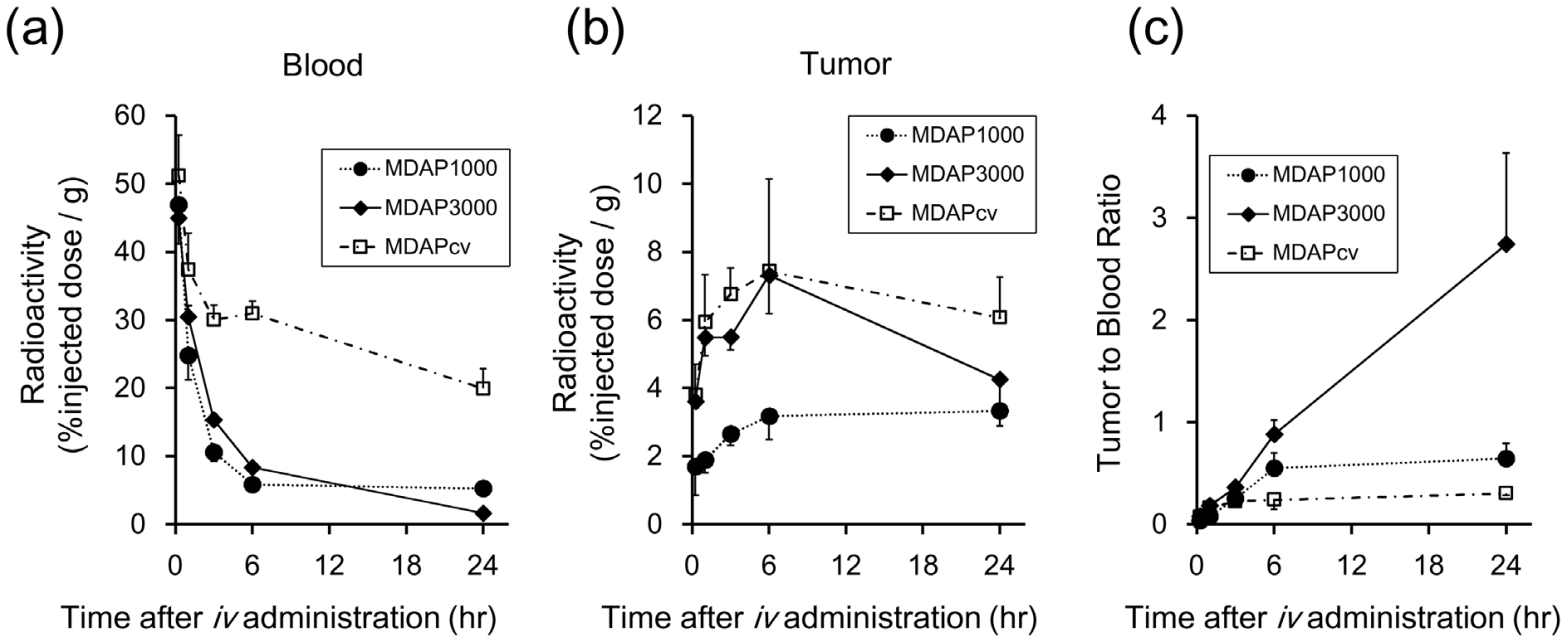
*In vivo* biodistribution experiment in HT1080-bearing mice with intravenous injection of MDAP_1000_, MDAP_3000_, and MDAP_CV_. (a) Time radioactivity curves in blood. (b) Time radioactivity curves in tumors. (c) Tumor to blood radioactivity ratios.

**Figure 5:**
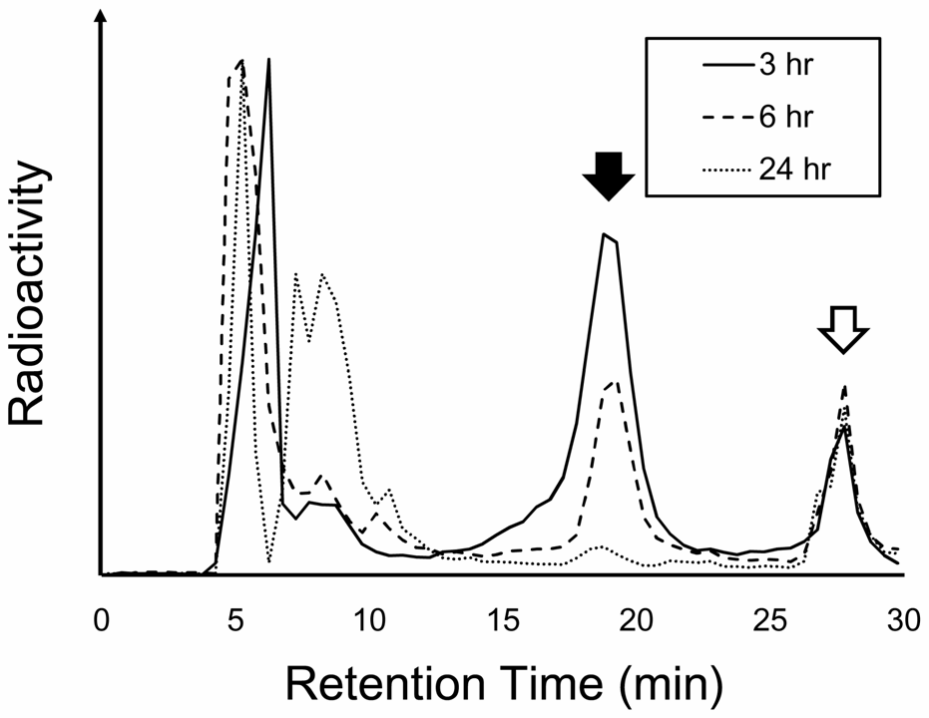
Reverse phase HPLC analysis of tumors excised 3, 6, and 24_3000_. Black and white arrows indicate the intact and cleaved peaks, respectively.

**Figure 6:**
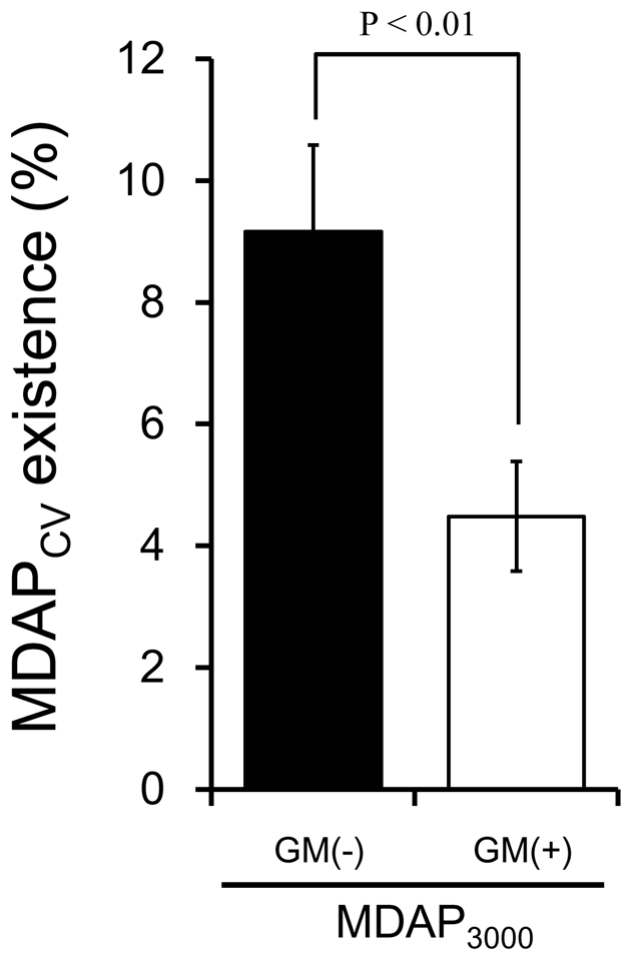
MDAP_CV_ (%) present in tumors after intratumoral injection of MDAP_3000_ with or without MMP inhibitor (GM6001).

**Figure 7:**
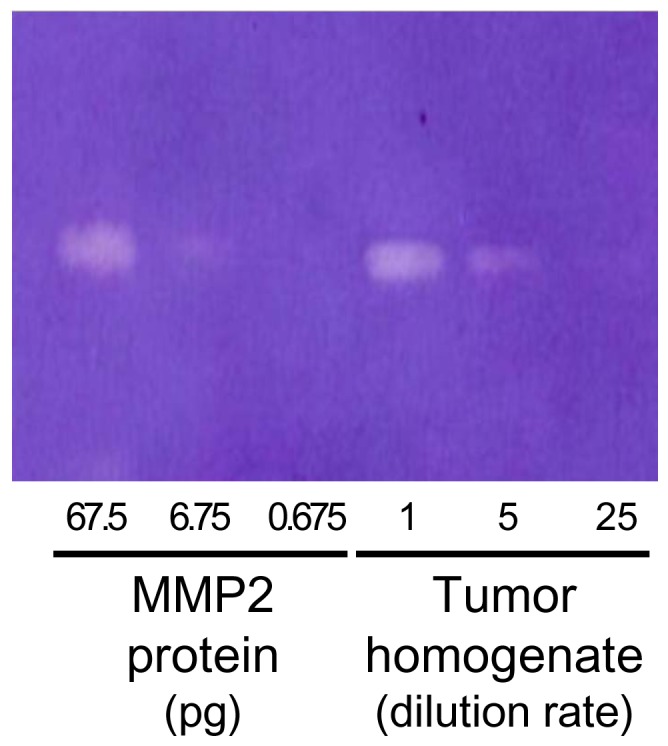
Zymography of recombinant MMP-2 protein and excised HT1080 tumor homogenates.

Radioactivity distribution profiles after intravenous administration of MDAP_1000_, MDAP_3000_ and MDAP_CV_ are shown in Tables 2, 3 and 4, respectively. To compare probe pharmacokinetics, changes in radioactivity in the blood and tumor are plotted as a function of time after administration (Fig. 4a, b), and as tumor to blood ratios (Fig. 4c). MDAP_3000_ exhibited rapid blood clearance (Fig. 4a) and high tumor accumulation (Fig. 4b). Thus, among the probes MDAP_3000_ achieved significantly higher tumor to blood ratios (2.74±0.89 at 24 hr, Table 3 and Fig. 4c). MDAP_1000_ also exhibited rapid blood clearance (Fig. 4a) but showed low tumor accumulation (Fig. 4b). Meanwhile, MDAP_CV_ showed a slow blood clearance (Fig. 4a) and the lowest tumor to blood ratios over the experimental period (0.30±0.02 at 24 hr, Table 4 and Fig. 4c). Pharmacokinetic analyses on whole body (Table 5) and tumors (Table 6) revealed the above points indicated by the biodistribution data more clearly. As shown in Table 5, MDAP_3000_ and MDAP_CV_ displayed similar distribution volumes while MDAP_CV_ showed slower total clearance as well as longer half-lives and mean residence time than MDAP_3000_. Table 6 shows an approximately seven-fold slower washout rate of MDAP_3000_ from the tumor (k2 = 1.2×10^−3^ min^−1^) compared to MDAP_CV_ (k2 = 8.6×10^−3^ min^−1^). In addition, HPLC analysis of tumors excised 3, 6 and 24 hr after intravenous injection of MDAP_3000_ showed a certain fraction of MDAP_CV_ existed in the tumor (Fig. 5, white arrow). The intact form of MDAP_3000_ indicated by the black arrow disappeared with time as shown in the HPLC chart. A certain fraction of MDAP_CV_ was also present inside the tumor after intratumoral injection of MDAP_3000_, which could be blocked by inhibitor treatment (Fig. 6). Other peaks observed around 5–10 min could represent further metabolites that are produced inside the cells. MMP-2 enzymatic activity of the tumor homogenate was confirmed by zymography (Fig. 7). The effective dose of MDAP_3000_ estimated from biodistribution data was 5.08×10^−2^ mSv/MBq.

**Table 2:** Radioactivity biodistribution after intravenous administration of MDAP_1000_ in tumor bearing mice.

	Time after administration (hr)				
	0.25	1	3	6	24
Blood	46.93±5.71	24.81±3.60	10.58±1.28	5.85±0.74	5.26±0.94
Heart	9.40±2.03	8.43±1.19	7.47±0.45	5.71±0.61	4.11±0.44
Lung	21.23±2.65	12.56±1.70	6.17±0.88	4.38±0.32	2.63±0.28
Liver	34.27±5.87	42.27±8.44	50.70±7.52	30.08±5.98	8.89±1.11
Kidneys	14.24±1.90	11.66±1.17	10.56±1.49	9.53±1.08	7.01±0.45
Stomach^a^	1.03±0.15	1.13±0.21	1.14±0.33	0.73±0.06	0.49±0.09
Intestine	4.18±0.58	8.97±0.28	20.39±4.51	13.23±3.92	4.90±0.90
Pancreas	4.28±0.41	5.52±1.05	4.50±1.45	5.53±1.77	6.21±3.76
Spleen	8.06±0.23	7.72±1.86	10.01±3.77	10.00±2.61	4.82±1.10
Muscle	2.15±0.57	1.94±0.33	2.00±0.38	1.68±0.46	1.55±0.13
Tumor	1.70±0.85	1.90±0.38	2.65±0.33	3.18±0.69	3.33±0.44
T/B	0.04±0.01	0.08±0.02	0.25±0.05	0.55±0.15	0.65±0.15

Data are presented as % injected dose per gram. Each value represents the mean ± s.d. for 3 animals at each interval. T/B means tumor to blood ratio.

a Presented as % injected dose per organ.

**Table 3:** Radioactivity biodistribution after intravenous administration of MDAP_3000_ in tumor bearing mice.

	Time after administration (hr)				
	0.25	1	3	6	24
Blood	44.98±2.53	30.48±1.67	15.33±0.81	8.33±0.58	1.61±0.27
Heart	9.26±0.86	8.20±0.74	6.70±0.59	5.64±0.79	3.78±0.39
Lung	20.44±4.77	15.92±0.58	9.72±1.02	5.56±0.75	2.50±0.44
Liver	18.83±1.38	18.45±0.81	22.83±3.72	17.61±2.53	10.51±0.89
Kidneys	11.85±0.84	10.60±0.38	11.89±0.76	11.40±1.18	10.81±2.35
Stomach^a^	0.48±0.04	0.69±0.21	1.17±0.82	0.97±0.46	0.54±0.07
Intestine	3.59±0.24	5.85±0.11	13.13±1.73	14.08±2.39	3.70±1.06
Pancreas	3.91±0.33	4.35±0.74	5.25±0.57	4.97±1.31	3.92±0.68
Spleen	6.53±0.34	5.65±0.98	5.41±0.64	4.94±1.51	3.92±0.24
Muscle	1.72±0.76	1.27±0.20	1.49±0.15	1.43±0.28	1.02±0.16
Tumor	3.60±0.17	5.49±0.53	5.49±0.36	7.31±1.12	4.25±0.79
T/B	0.08±0.01	0.18±0.03	0.36±0.04	0.88±0.14	2.74±0.89

Data are presented as % injected dose per gram. Each value represents the mean ± s.d. for 3 animals at each interval. T/B means tumor to blood ratio.

a Presented as % injected dose per organ.

**Table 4:** Radioactivity biodistribution after intravenous administration of MDAP_CV_ in tumor bearing mice.

	Time after administration (hr)				
	0.25	1	3	6	24
Blood	51.19±6.00	37.43±5.29	30.07±2.16	30.99±1.77	19.94±2.95
Heart	9.08±1.72	8.04±0.37	7.09±0.12	6.61±1.43	4.84±1.43
Lung	25.41±7.86	18.25±2.62	15.52±3.02	13.07±3.55	9.08±0.75
Liver	13.05±1.68	11.57±2.33	8.63±0.32	8.47±1.31	7.13±0.54
Kidneys	10.48±1.00	8.84±1.71	7.99±0.42	7.83±1.55	6.91±0.70
Stomach^a^	0.60±0.02	0.53±0.23	0.81±0.40	0.83±0.37	0.43±0.13
Intestine	2.02±0.23	3.37±0.48	6.13±3.17	14.17±10.09	25.12±5.34
Pancreas	2.21±0.45	2.61±0.42	3.17±0.11	2.56±0.94	2.22±0.45
Spleen	6.20±0.62	4.96±0.40	4.12±0.57	4.45±0.45	4.61±0.22
Muscle	1.07±0.17	1.17±0.14	1.44±0.24	1.50±0.48	1.53±0.53
Tumor	3.80±0.90	5.94±1.39	6.76±0.77	7.43±2.71	6.08±1.19
T/B	0.08±0.02	0.16±0.05	0.22±0.01	0.24±0.10	0.30±0.02

Data are presented as % injected dose per gram. Each value represents the mean ± s.d. for 3 animals at each interval. T/B means tumor to blood ratio.

a Presented as % injected dose per organ.

**Table 5:** Whole body pharmacokinetic parameters.

	Blood half-life (min)		Distribution volume (ml)	Mean residence time (min)	Clearance (ml/min)
	Fast	Slow			
MDAP_1000_	4.0×10	2.6×10^3^	1.71	2.1×10^2^	8.3×10^−3^
MDAP_3000_	5.6×10	4.3×10^2^	1.94	2.5×10^2^	7.9×10^−3^
MDAP_CV_	2.5×10^2^	5.4×10^15^	2.16	8.6×10^2^	2.5×10^−3^

**Table 6:** Probe pharmacokinetic parameters between blood and tumors.

	K1		k2	
	(ml/g/min)		(1/min)	
	Mean	%SE	Mean	%SE
MDAP_1000_	7.1×10^−4^	1.14	1.1×10^−3^	2.11
MDAP_3000_	1.3×10^−3^	4.09	1.2×10^−3^	6.98
MDAP_CV_	2.2×10^−3^	8.2	8.6×10^−3^	7.82

Data were calculated by PMOD ver. 3.2 (1-tissue compartment model) with biodistribution results. K1 and k2 are rate constants for transfer from blood to tumor and clearance from tumor to blood, respectively.

## Discussion

In this study we tested the feasibility and usefulness of our novel drug design strategy wherein the MMP-2 activity-dependent anchoring probe MDAP is used for MMP-2 activity imaging in cancer. Both *in vitro* and *in vivo* experiments indicated that MDAP_3000_ displayed cell membrane anchoring properties following substrate cleavage by MMP-2 in tumors, which led to a somewhat slower washout of radioactivity from tumors. As such, we successfully demonstrated the possible application of our MDAP strategy for tumor imaging.

During the initial stages of probe design, we selected a double alkyl chain as the anchoring unit based on a previous report indicating that double alkyl chains were superior to single alkyl chains for cell membrane anchoring. The length of the double alkyl chain (palmitoyl group and 10-amino-decanoic acid in the probe structures) was selected based on results from a preliminary cell binding experiment. We then combined other moieties to fulfill the requirements for the MDAP. Specifically, ^111^In was selected for radiolabeling due to its simple and rapid radiosynthesis with a hydrophilic chelating agent (DTPA)-conjugated precursor under mild conditions [9]. Among previously reported MMP-2 substrate peptides, Pro-Leu-Gly-Val-Arg-Gly was selected so that the amino terminus residues (Pro-Leu-Gly) would retain their radioactivity after cleavage and not inhibit the anchoring property of the double alkyl chain [10]. PEG was selected because of the hydrophilicity and steric hindrance that would allow it to function as an inhibitor of the MDAP probes and three PEG molecule types (MW: 1000, 3000, and 5000) were tested to determine the optimum molecular weight both in terms of inhibitive capacity on anchoring and resistance to MMP-2 cleavage. As a result, we successfully obtained radiolabelled MDAP probes with a high radiochemical yield and purity that allowed their use in *in vitro* and *in vivo* experiments without additional purification.

PEG is often used to modify molecular probes to alter their hydrophilicity and pharmacokinetics [11], [12]. Therefore, we expected both the hydrophilicity and steric hindrance of PEG to alter the properties of MDAP. Cellular accumulation results clearly divided the probes into a high accumulation group (MDAP_1000_ and MDAP_CV_) and low accumulation group (MDAP_3000_ and MDAP_5000_) where the probe hydrophilicity gradually increased with the molecular weight of PEG as shown in the obtained log P values. As such, the steric hindrance rather than the hydrophilicity imparted by PEG functioned as an inhibitor of the anchoring probe moiety. While MDAP_1000_, MDAP_3000_, and MDAP_5000_ were cleaved by MMP-2 as expected, the cleavability of MDAP_5000_ was rather low, which indicated that PEG located around the probe inhibited its interactions with other molecules, including the MMP-2 protein. Thus, the *in vitro* experiments showed that MDAP_3000_ was recognized as a possible suitable probe for further *in vivo* evaluation.

The *in vivo* biodistribution study revealed a rapid blood clearance and high tumor uptake of MDAP_3000_ after intravenous injection, while MDAP_CV_ showed high radioactivity in both blood and tumors. Thus, a significantly higher tumor to blood radioactivity ratio (T/B ratio), an imaging index [9], was obtained for the MDAP_3000_ group, indicating the superior characteristics of MDAP_3000_ as an imaging agent *in vivo*. The effective dose of MDAP_3000_ estimated from the biodistribution data was acceptable for the use of MDAP_3000_ as an imaging agent [13]. On the other hand, MDAP_CV_ showed high radioactivity remaining in the blood, which implied nonspecific binding with serum proteins and/or blood cells. This result suggested that intravenously administered MDAP_CV_ may bind with serum proteins and exist within the interstitial space of tumors as the complex form. This possibility was also indicated by MDAP_CV_ having the lowest T/B ratios (less than 0.3) over the experimental period. The MDAP_CV_ biodistribution results indicated that MDAP_CV_ generated in other tissues after systemic MDAP administration would be less frequently redistributed to tumors. Thus, tumor accumulation of MDAP_3000_ was not derived from redistribution of cleaved MDAP_CV_ since MDAP_3000_ showed rapid blood clearance and increase in the T/B ratio with time after intravenous administration. Nevertheless, there was a measurable percentage of MDAP_CV_ existing in tumors after intravenous and intratumoral injections of MDAP_3000_, and MDAP_CV_ resulting from intratumoral injection of MDAP_3000_ was somewhat decreased by the MMP inhibitor. MDAP_CV_ could be generated in the tumor as a result of MDAP_3000_ cleavage by MMP-2. HPLC analyses on excised tumors also indicate accumulation and retention of MDAP_3000_ followed by gradual degradation to hydrophilic metabolites in tumors. It is quite reasonable from the MDAP strategy that pharmacokinetic analysis provided a considerably slower washout rate (k2 value) for MDAP_3000_ compared to MDAP_CV_, because the slower washout rate for MDAP_3000_ indicates MDAP_3000_ radioactivity capture in tumors. Regarding the limitations of this study, direct evidence that MDAP_3000_ was anchored in the cell membrane after MMP-2 activation in tumor tissues was not obtained. To evaluate whether the MDAP probe is anchored in the cell membrane after MMP-2 activation more precisely, further studies such as HPLC analysis of membrane fractions from the tumor cells alone or confocal microscopy approaches using an appropriate fluorophore introduced into MDAP_3000_ are needed. However, the *in vitro* and *in vivo* studies did show MMP-2 dependent probe cleavage, retention of radioactivity in tumor tissues and slow washout from tumor tissues, indicating the possible application of the MDAP strategy.

Although fluorescence activatable probes have been reported for MMP imaging [14]-[17], nuclear medical imaging has great potential for *in vivo* applications because it can quantitatively detect signals from tissues deep in the body but also can be applied for use in cancer therapies involving α, β, or auger electron emitters [18], [19]. Since MDAP_3000_ might allow selective readout of the activated MMP-2 subpopulation due to signal amplification as compared to MMP binding probes, MDAP_3000_ could be a potential lead compound for use in future applications. Further studies to compare MDAP_3000_ with other radiolabeled MMP imaging probes are thus warranted [20], [21].
